# Expression of functional inhibitory neurotransmitter transporters GlyT1, GAT-1, and GAT-3 by astrocytes of inferior colliculus and hippocampus

**DOI:** 10.1186/s13041-018-0346-y

**Published:** 2018-01-25

**Authors:** Elsa Ghirardini, Simon L. Wadle, Vanessa Augustin, Jasmin Becker, Sina Brill, Julia Hammerich, Gerald Seifert, Jonathan Stephan

**Affiliations:** 1 Animal Physiology Group, Department of Biology, University of Kaiserslautern, Erwin Schroedinger-Strasse 13, D-67663 Kaiserslautern, Germany; 2 Department of Medical Biotechnology and Translational Medicine, University of Milan, via Vanvitelli 32, I-20129 Milan, Italy; 3 Pharmacology and Brain Pathology Lab, Humanitas Clinical and Research Center, via Manzoni 56, I-20089 Rozzano, Italy; 4 Institute of Cellular Neurosciences, Medical Faculty, University of Bonn, Sigmund-Freud-Strasse 25, D-53105 Bonn, Germany

**Keywords:** Inferior colliculus, Hippocampus, GlyT1, GAT-1, GAT-3

## Abstract

Neuronal inhibition is mediated by glycine and/or GABA. Inferior colliculus (IC) neurons receive glycinergic and GABAergic inputs, whereas inhibition in hippocampus (HC) predominantly relies on GABA. Astrocytes heterogeneously express neurotransmitter transporters and are expected to adapt to the local requirements regarding neurotransmitter homeostasis. Here we analyzed the expression of inhibitory neurotransmitter transporters in IC and HC astrocytes using whole-cell patch-clamp and single-cell reverse transcription-PCR. We show that most astrocytes in both regions expressed functional glycine transporters (GlyTs). Activation of these transporters resulted in an inward current (*I*_Gly_) that was sensitive to the competitive GlyT1 agonist sarcosine. Astrocytes exhibited transcripts for GlyT1 but not for GlyT2. Glycine did not alter the membrane resistance (*R*_M_) arguing for the absence of functional glycine receptors (GlyRs). Thus, *I*_Gly_ was mainly mediated by GlyT1. Similarly, we found expression of functional GABA transporters (GATs) in all IC astrocytes and about half of the HC astrocytes. These transporters mediated an inward current (*I*_GABA_) that was sensitive to the competitive GAT-1 and GAT-3 antagonists NO711 and SNAP5114, respectively. Accordingly, transcripts for GAT-1 and GAT-3 were found but not for GAT-2 and BGT-1. Only in hippocampal astrocytes, GABA transiently reduced *R*_M_ demonstrating the presence of GABA_A_ receptors (GABA_A_Rs). However, *I*_GABA_ was mainly not contaminated by GABA_A_R-mediated currents as *R*_M_ changes vanished shortly after GABA application. In both regions, *I*_GABA_ was stronger than *I*_Gly_. Furthermore, in HC the *I*_GABA_/*I*_Gly_ ratio was larger compared to IC. Taken together, our results demonstrate that astrocytes are heterogeneous across and within distinct brain areas. Furthermore, we could show that the capacity for glycine and GABA uptake varies between both brain regions.

**Electronic supplementary material:**

The online version of this article (10.1186/s13041-018-0346-y) contains supplementary material, which is available to authorized users.

## Introduction

Glycine and GABA mediate synaptic inhibition in matured circuits. Glycinergic and GABAergic inhibition are attributed predominantly to caudal and rostral brain regions, respectively. Nonetheless, both neurotransmitters coexist throughout the whole brain.

Mixed inhibitory synaptic transmission, with co-release of glycine and GABA from the same presynaptic terminal, takes place in various caudal brain regions, such as auditory brainstem, ventral respiratory group, cerebellum, and spinal cord [1–8]. In more rostral brain regions, like the hippocampus (HC), GABA is utilized for inhibitory synaptic transmission [9, 10], while glycine co-released from glutamatergic terminals can modulate NMDA receptor (NMDAR)-mediated signaling [11, 12]. Accordingly, glycine transporters (GlyTs) and GABA transporters (GATs) are widely expressed in astrocytes and neurons [13–16] to enable neurotransmitter clearance, reuptake, and modulation of neuronal signaling [15, 17, 18]. Astrocytes mainly express GlyT1 (*Slc6A9*), GAT-1 (*Slc6A1*), and/or GAT-3 (*Slc6A11*), which mediate an inward current and concomitant depolarization [19]. In addition, astrocytes can express ionotropic receptors for glycine (GlyRs) and GABA (GABA_A_Rs) [20–26].

**Fig. 1:**
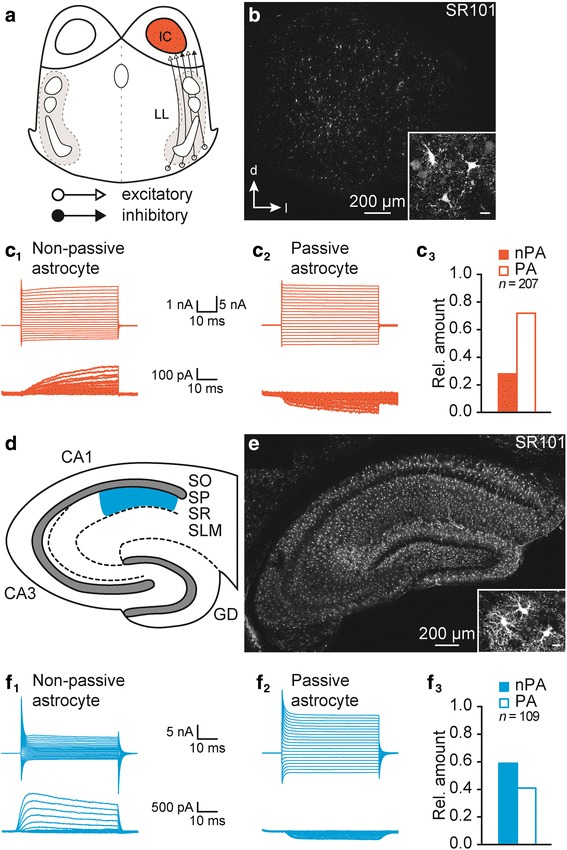
Identification of IC and HC astrocytes. **a**, **d**: Semischematic drawings of coronal sections containing the central nucleus of the IC together with the lateral lemniscal tract (LL) (*A*) and the CA1 region of the HC (CA: *cornu ammonis*; GD: *gyrus dentatus*; SO: *stratum oriens*; SP: *stratum pyramidale*; SR: *stratum radiatum*; SLM: *stratum lacunosum moleculare*). **b**, **e**: SR101-labeled cells (astrocytes) were widely distributed within the IC (b) and the HC (e). They exhibited a small soma and were highly branched (insets in b and e; scale bar: 10 μm). **c**, **f**: Astrocytes were clamped to *E*_H_ = −85 mV and were stepwise hyper- and depolarized from −150 mV to +50 mV, with 10 mV increments. Membrane currents were recorded (top) and leak currents were subtracted (p/4; bottom). Non-passive astrocytes (nPA) expressed time- and voltage-dependent outward currents (c_1_, f_1_), whereas passive astrocytes (PA) lacked these currents (c_2_, f_2_). About 2/3 of IC astrocytes were passive (c_3_), whereas it was the opposite in the HC (f_3_). *n* represents the number of recorded cells

In a previous study, we analyzed the expression of functional GlyTs and GATs in astrocytes in the lateral superior olive (LSO) – a conspicuous auditory brainstem center whose main inhibitory input is glycinergic after early postnatal development [2, 3]. Astrocytes in this nucleus express functional GlyT1, GAT-1, and GAT-3 [19]. To study the region-dependent heterogeneity of GlyT and GAT expression in astrocytes, we chose two systems that contrast the LSO with respect to the utilization of glycine and GABA for inhibitory synaptic transmission: 1) The inferior colliculus (IC) residing in the midbrain belongs to the rostral part of the auditory brainstem and serves as a major hub for processing auditory cues [4, 27]. Afferents from all auditory brainstem centers converge in the lateral lemniscal tract (LL) and project to the IC (Fig. 1a) [4, 8, 28]. The inhibitory part of the tract consists of glycinergic and GABAergic projections [8, 29–31]. Accordingly, IC astrocytes can be proposed to express GlyTs and GATs to account for neurotransmitter uptake. GlyT1 expression was found in the IC and attributed to glial cells [11, 13, 32]. Likewise, GAT-1 and GAT-3 are present in the IC [33, 34]. However, GlyTs and GATs in IC astrocytes have not yet been electrophysiologically characterized. 2) The HC is the second system of interest. Whereas its main circuitry is glutamatergic [35, 36], inhibitory synaptic transmission arises from GABAergic interneurons [9, 10]. In line with this, astrocytes in the *stratum radiatum* express GAT-3, whereas GAT-1 has been attributed to interneurons [21, 37]. Glycine is co-released from glutamatergic terminals and modulates NMDAR-mediated signaling [11, 12]. For uptake of released glycine, GlyT1 is expressed in astrocytes and presynaptic terminals [11, 38–40]. However, functionality of GlyT1 in HC astrocytes has not been demonstrated prior to this study.

Here we analyzed the heterogeneity of expression and function of inhibitory neurotransmitter transporters in astrocytes from IC and HC. Using whole-cell patch-clamp recordings from sulforhodamine 101 (SR101)-labeled astrocytes [19, 41, 42] and concomitant application of glycine or GABA to provoke transporter activation, together with single-cell reverse transcription (RT)-PCR, our results demonstrate that all IC astrocytes and about half of the HC astrocytes expressed functional GlyT1, GAT-1, and GAT-3. In contrast, GlyT2, GAT-2, and BGT-1 were never found. From our experiments, we can exclude that transporter currents were contaminated by respective ionotropic receptor-mediated currents. As expected, GAT activity was much stronger in HC astrocytes compared to IC astrocytes. Concurrently, our results show that IC and HC astrocytes exhibit heterogeneous properties, which reflect region-specific adaptation to local circuitry.

## Methods

### Preparation of acute tissue slices and labeling of astrocytes

We used tissue from C57BL/6 wild type mice of both genders at postnatal days 10-12 for our experiments. Mice were treated in accordance with the German law for conducting animal experiments and the NIH guidelines for the care and use of laboratory animals. Acute coronal slices were retrieved from midbrain and forebrain containing IC and HC, respectively. After decapitation, the brain was quickly transferred into ice-cold cutting solution containing (in mM): 26 NaHCO_3_, 1.25 NaH_2_PO_4_, 2.5 KCl, 1 MgCl_2_, 2 CaCl_2_, 260 D-glucose, 2 Na-pyruvate, and 3 myo-inositol, pH 7.4, bubbled with carbogen (95% O_2_, 5% CO_2_). 270 μm thick slices were cut using a vibratome (VT1200 S, Leica). Thereafter, slices were transferred to artificial cerebrospinal fluid (ACSF) containing (in mM): 125 NaCl, 25 NaHCO_3_, 1.25 NaH_2_PO_4_, 2.5 KCl, 1 MgCl_2_, 2 CaCl_2_, 10 D-glucose, 2 Na-pyruvate, 3 myo-inositol, and 0.44 ascorbic acid, pH 7.4, bubbled with carbogen. Slices were incubated for 30 min at 37 °C in 0.5-1 μM SR101 and washed for another 30 min at 37 °C in SR101-free ACSF. This resulted in reliable labeling of astrocytes as shown before [19, 41]. Thereafter, slices were kept at room temperature (20-24 °C). All chemicals were purchased from Sigma-Aldrich or AppliChem, if not stated otherwise.

### Electrophysiology

Whole-cell patch-clamp experiments were done as described before [19]. Briefly, the recording chamber was placed at an upright microscope equipped with infrared differential interference contrast (Eclipse FN1, Nikon, 60× water immersion objective, N.A. 1.0) and an infrared video camera (XC-ST70CE, Hamamatsu). Voltages and currents were recorded using a double patch-clamp EPC10 amplifier and PatchMaster software (HEKA Elektronik). The patch pipettes were pulled from borosilicate glass capillaries (GB150(F)-8P, Science Products) using a horizontal puller (P-87, Sutter Instruments). Pipettes had a resistance of 3-7 MΩ using an intracellular solution containing (in mM): 140 K-gluconate, 5 EGTA (glycol-bis(2-aminoethylether)-*N*,*N*′,*N*′,*N*′-tetraacetic acid), 10 Hepes (N-(2-hydroxyethyl)piperazine-N′-2-ethanesulfonic acid), 1 MgCl_2_, 2 Na_2_ATP, and 0.3 Na_2_GTP, pH 7.30. In some experiments the intracellular solution contained biocytin (0.3%, Biomol) or alexa fluor (AF) 568 (100 μM, Invitrogen) to allow the postfixational reconstruction of IC and HC neurons, respectively. Biocytin was labeled with NeutrAvidin-horseradish peroxidase conjugate (1:1000; Invitrogen) [43].

Astrocytes and neurons in the central nucleus of the IC (Fig. 1a; Additional file 1: Figure S1*A*_*1*_) and CA1 region of the HC (Fig. 1d; Additional file 1: Figure S1*A*_*2*_) were clamped to a holding potential (*E*_H_) of −85 mV and −70 mV, respectively. The cells were hyper- and depolarized using a standard step protocol ranging from −150 to +50 mV with 10 mV increments. The resulting current traces were sampled at 50 kHz. We performed a standard leak subtraction protocol (p/4) to isolate currents mediated by voltage-activated channels. Four step protocols were executed repetitively that comprised a reduced step size of 25%. Thereafter, the recorded current traces were add together and subtracted from the initial recording (Fig. 1c_1-2_, f_1-2_, Additional file 1: Figure S1*B*).

**Fig. 2:**
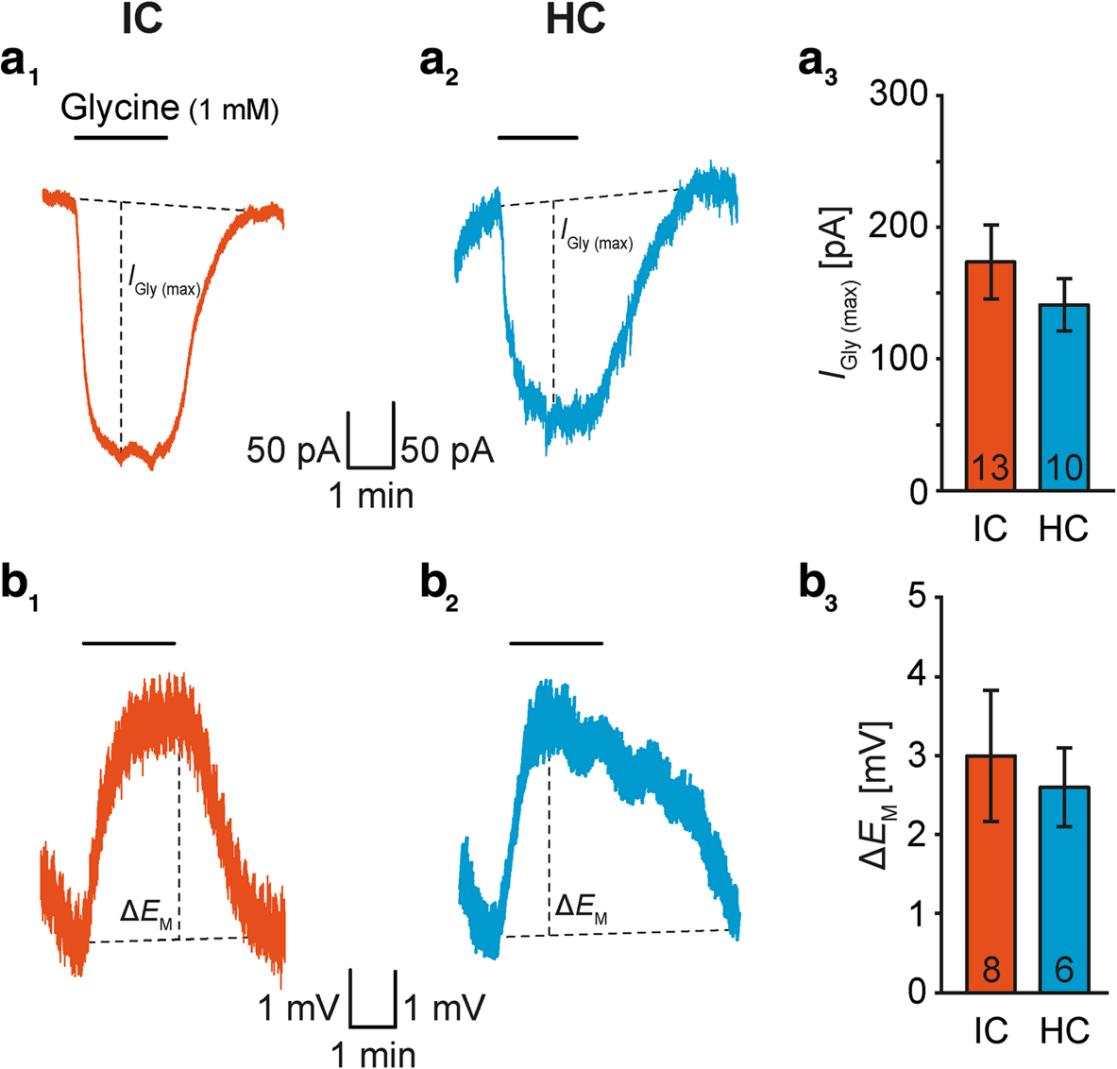
Glycine-induced transients in IC and HC astrocytes. **a**-**b**: Application of glycine (1 mM) caused an inward current (*I*_Gly (max)_; *E*_H_ = −85 mV; a_1-2_) and a membrane depolarization (Δ*E*_M_; b_1-2_), which was not different between IC and HC astrocytes (a_3_, b_3_). The number of recorded cells (*n*) is given within the diagrams. Shown are mean values ± SEM. Significance levels in panels a_3_ and b_3_ were Šidák corrected for two comparisons (see Methods section)

**Fig. 3:**
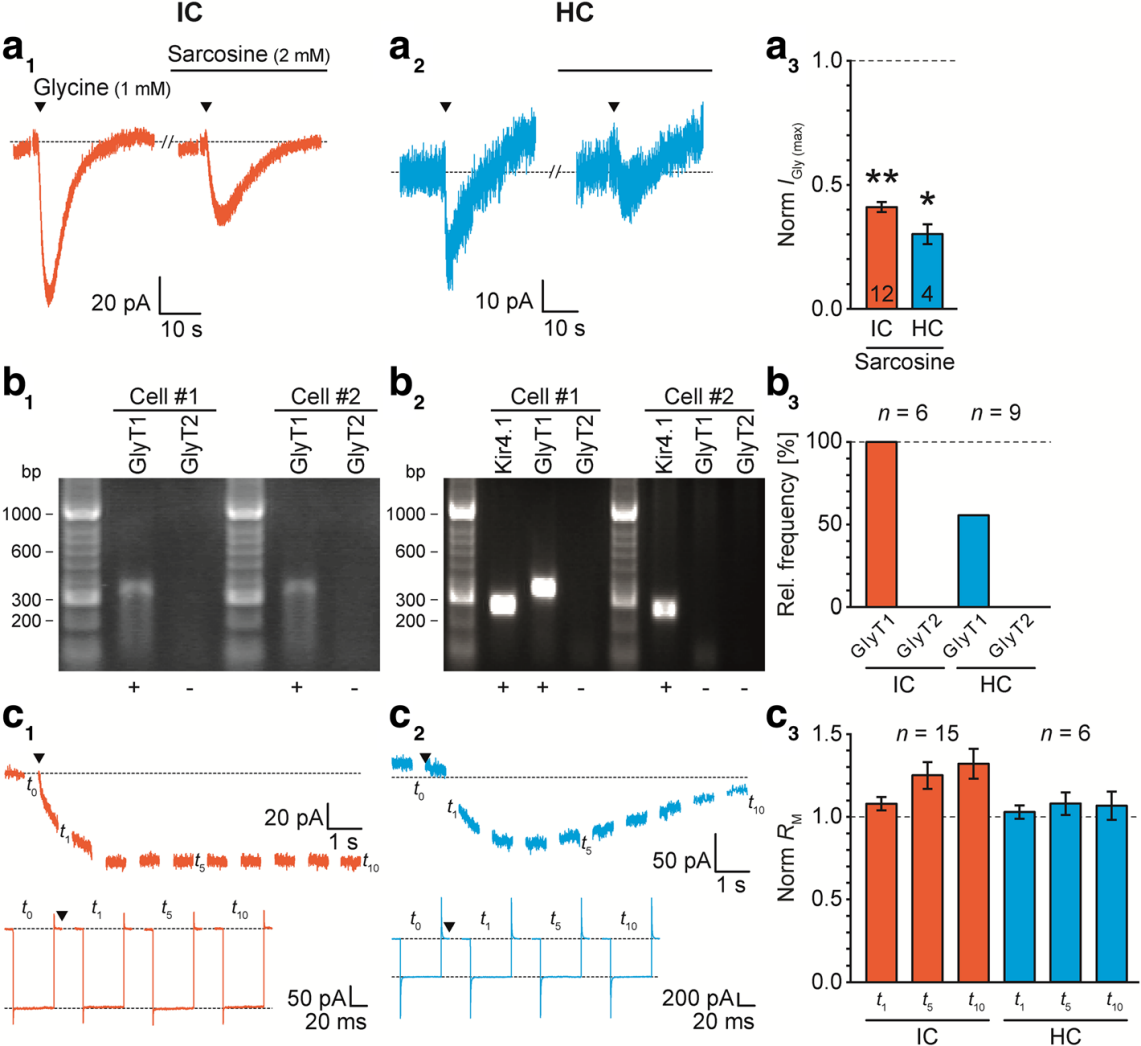
IC and HC astrocytes express GlyT1. **a:** Focal application of glycine (1 mM, 0.5 s; triangles) elicited an *I*_Gly_ (*E*_H_ = −85 mV; a_1-2_). The competitive GlyT1 agonist sarcosine (2 mM) reduced *I*_Gly (max)_ (a_3_). **b:** Single-cell RT-PCR revealed transcripts for GlyT1 in all IC astrocytes and about half of the HC astrocytes. Transcripts for GlyT2 were not found. **c:** Focal application of glycine (1 mM, 10 s; triangle) induced a long lasting inward current (*E*_H_ = −85 mV; c_1-2_, top). Responses to test pulse (see Additional file 2: Figure S2*A*) were used to calculate *R*_M_ at *t*_0_, *t*_1_, *t*_5_, and *t*_10_ (c_1-2_, bottom). Upon glycine application, *R*_M_ was not reduced in IC and HC astrocytes (c_3_). The number of recorded cells (*n*) is given within the diagrams. Shown are mean values ± SEM. Significance levels in c_3_ were Šidák corrected for three comparisons (see Methods section). bp: base pairs

**Fig. 4:**
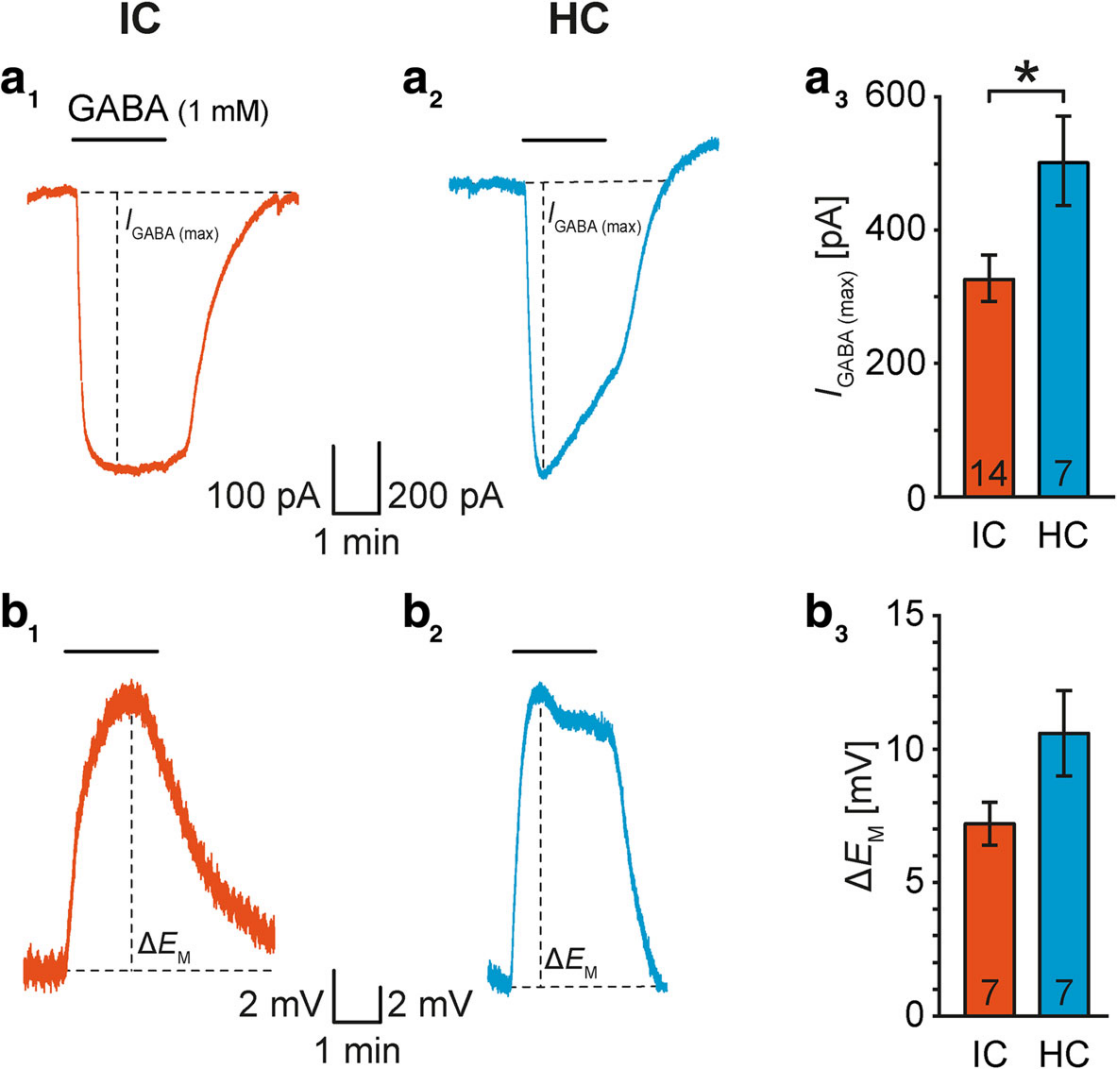
GABA-induced transients in IC and HC astrocytes. **a:** Application of GABA (1 mM) caused an inward current (*I*_GABA (max)_; *E*_H_ = −85 mV; a_1-2_), which was larger in HC astrocytes (a_3_). **b:** Similarly, GABA induced a membrane depolarization (Δ*E*_M_; b_1-2_), which was not significantly different between IC and HC astrocytes (b_3_). Shown are mean values ± SEM. Significance levels in panel a_3_ and b_3_ were Šidák corrected for two comparisons (see Methods section)

**Fig. 5:**
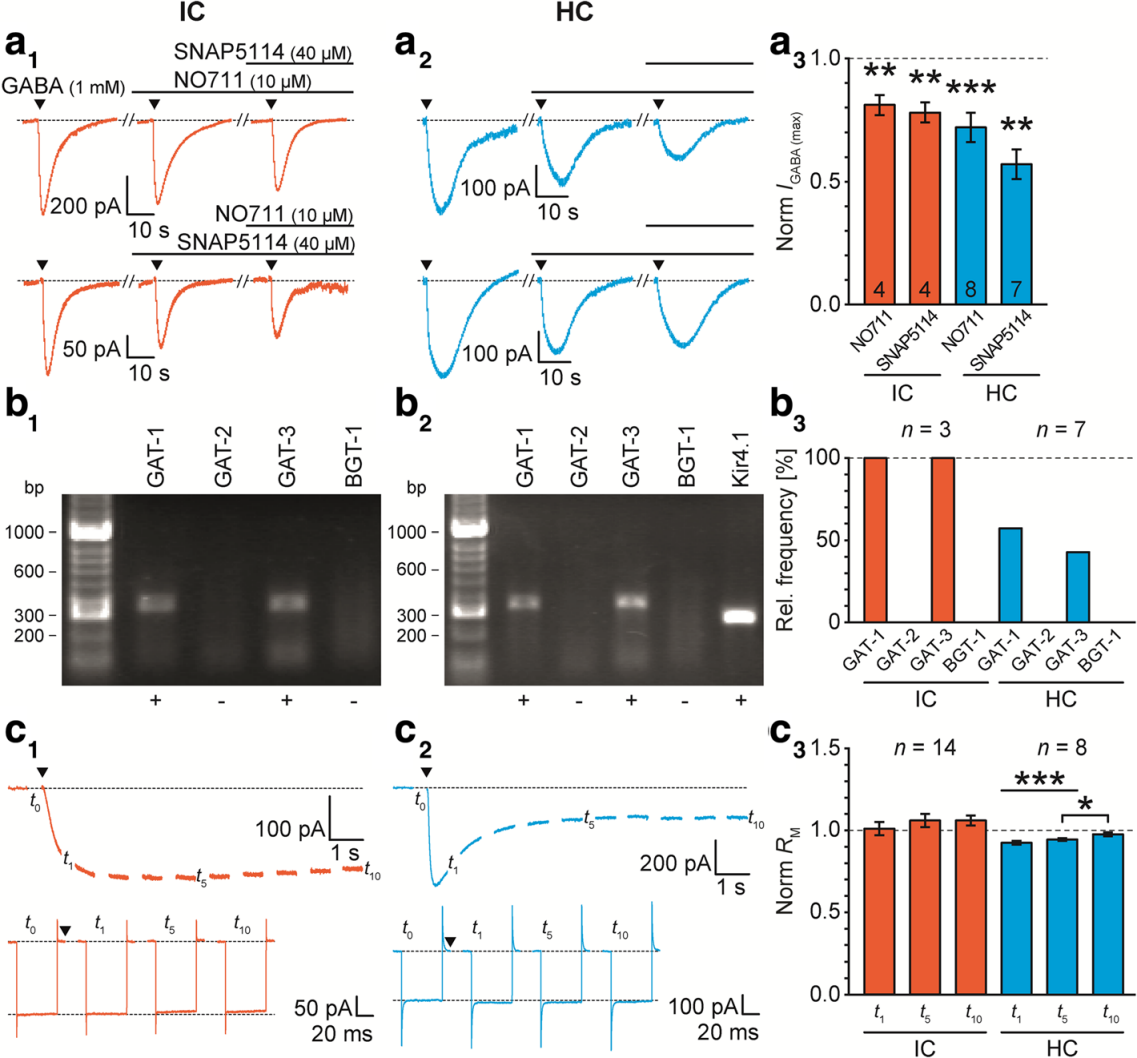
IC and HC astrocytes express GAT-1 and GAT-3. **a:** Focal application of GABA (1 mM, 0.5 s; triangles) elicited an *I*_GABA_ (*E*_H_ = −85 mV; a_1-2_). The non-competitive antagonists for GAT-1 (NO711, 10 μM) and GAT-3 (SNAP5114, 40 μM) reduced *I*_GABA (max)_ (a_3_). **b:** Single-cell RT-PCR revealed transcripts for GAT-1 and GAT-3 in all IC astrocytes and about half of the HC astrocytes. Transcripts for GAT-2 and BGT-1 were never present. **c:** Focal application of GABA (1 mM, 10 s; triangle) induced a long lasting inward current (*E*_H_ = −85 mV; c_1-2_, top). Responses to test pulse (see Additional file 2: Figure S2*A*) were used to calculate *R*_M_ at *t*_0_, *t*_1_, *t*_5_, and *t*_10_ (c_1-2_, bottom). Upon GABA application, *R*_M_ was transiently reduced exclusively in HC astrocytes (c_3_). The number of recorded cells (*n*) is given within the diagrams. Shown are mean values ± SEM. Significance levels in panel c_3_ were Šidák corrected for three comparisons (see Methods section). bp: base pairs

Glycine and GABA, both 1 mM in ACSF, were applied in two ways: 1) In experiments analyzing the maximal neurotransmitter-induced current and depolarization (Figs. 2 and 4) both transmitters were administered using a peristaltic pump (Reglo, Ismatec) at a rate of 1-2 ml/min. Data were sampled at 100 Hz. We monitored putative changes of membrane resistance (*R*_M_) and series resistance (*R*_S_) every 30 s (≙ 0.033 Hz) using hyperpolarizing test pulses (Δ*U* = 5 mV) [44]. The resulting currents were sampled at 20 kHz. 2) When pharmacologically isolating transporters mediating membrane currents (Figs. 3a and 5a) neurotransmitters were applied via focal pressure injection (PDES-2 T, NPI; 12 psi). Therefore, a pipette with a resistance of 3-7 MΩ was filled with glycine or GABA and positioned approximately 20 μm apart from the recorded cell [19]. Membrane currents were sampled at 1 kHz. In order to detect additionally short-lasting receptor-mediated changes in membrane conductance during focal application of neurotransmitters (Figs. 3c and *5*c, Additional file 2: Figure S2*B-E*), hyperpolarizing test pulses were applied at 1 Hz (Additional file 2: Figure S2*A*) and *R*_M_ of astrocytes and neurons was calculated [44]. All recordings were low-pass filtered at 2.9 kHz. Data were processed and analyzed using “IGOR Pro 6.2” software (Wavemetrics). Measurements were rejected if *R*_S_ exceeded 15 MΩ.

### Single-cell RT-PCR

The patch pipette was filled with 3 μl of intracellular solution. Next, astrocytes were patch-clamped as described in the preceding paragraph. After determination of the *I-V* relationship, the cytoplasm was sucked into the patch pipette, which was then retracted from the slice. The remaining cell parts were sucked into the patch pipette and the intracellular solution containing the cytoplasm was put into a 50 μl PCR reaction tube containing 3 μl of diethyl pyrocarbonate (0.1%)-treated water (ThermoFisher Scientific). To avoid degradation by RNAse activity the sample was immediately frozen in liquid nitrogen and stored at −80 °C. Samples were rejected, if the patch was unstable during cell extraction or fragments from neighboring cells stuck at the pipette.

For transcription of mRNA into cDNA reverse transcriptase (SuperScript III, 100 U; ThermoFisher Scientific), RNAse inhibitor (RNAseOUT, 40 U; ThermoFisher Scientific), random hexamers (50 μM, ThermoFisher Scientific), first-strand buffer (ThermoFisher Scientific), and dithiothreitol (DTT; 10 mM; ThermoFisher Scientific) were added to the frozen sample (total volume: 13 μl). Next, RT was performed for 1 h at 37 °C. Subsequently, a multiplex PCR was performed to identify transcripts of inhibitory neurotransmitter transporters. MPprimer software [45] was used to create primer sequences (Table 1). Primers were chosen to be located on different exons. Thus, amplification of DNA, which contains exons and introns, would result in larger product length compared to the amplicon of spliced mRNA that could be distinguished after gel electrophoresis. The PCR reaction mix contained: 5× PCR buffer including dNTPs (50 μM; Bioline), Taq Polymerase (4 U, Bioline), 200 nM primers (Eurofins Scientific), 10 μl of the RT reaction product, H_2_O (*ad* 50 μl, Ampuwa, Fresenius Kabi). Fifty PCR cycles were performed: denaturation for 25 s at 94 °C, annealing for 2 min (first 5 cycles) and 45 s (subsequent 45 cycles) at 51 °C, and elongation for 25 s at 72 °C. Afterwards, a second PCR with nested primers and 40 cycles was conducted: denaturation for 25 s at 94 °C, annealing for 2 min (first 5 cycles) and 45 s (subsequent 35 cycles) at 54 °C, and elongation for 25 s at 72 °C. The second PCR reaction mix contained Platinum Taq Polymerase (1 U, ThermoFisher Scientific), 10× PCR buffer (MgCl_2_-free; ThermoFisher Scientific), 2.5 mM MgCl_2_ (ThermoFisher Scientific), 50 μM dNTPs (Bioline), nested primers (200 nM, Eurofins Scientific), and 2 μl of the first PCR reaction product.

**Table 1:** Primers for single-cell RT-PCR

Gene	Sequence	Expected amplicon size [bp]	GeneBank accession number
GlyT1	fwd 5’-CACCAACTGTGCTACCAGCGTCTA-3′ rev 5’-GCATAGTTGTCCATCAGCAGCAGC-3’	390	NM_008135.4
GlyT1 (nested)	fwd 5’-GCTTCGTCATCTTCTCCATCCTG-3′ rev 5’-CAGTAGATGCCTGCCTGGCTG-3’	337	
GlyT2	fwd 5’-TCCTGTGTTATCGGTGACCATCC-3′ rev 5’-GAGTGGCCGCATCCTTCCATA-3’	430	NM_148931.3
GlyT2 (nested)	fwd 5’-TCTGCATGACTGCCTATCCGAACT-3′ rev 5’-TGTGATGAAGTACCAGATGCCGG-3’	329	
GAT-1	fwd 5’-GATGACAGATGGACTGGACAAG-3′ rev 5’-CACGATGGAGAAGATGACGAAT-3’	430	NM_178703.4
GAT-1 (nested)	fwd 5‘-TTGGACTGGAAAGGTGGTCTA-3‘ rev 5’-ACGATGGAGAAGATGACGAATC-3’	324	
GAT-2	fwd 5’-TTATTGTGTCCGTCATCTCGTT-3′ rev 5’-ACTTCTTGTTGTAGGTCAGTGG-3’	309	NM_144512.2
GAT-2 (nested)	fwd 5’-GTTCTTCATCGGGCTCATCAT-3′ rev 5’-TAGGTCAGTGGCGTGTATTTG-3’	279	
GAT-3	fwd 5’-GGGCATCTTCATCTTCTTTCTG-3′ rev 5’-GGTTCAGGATTCATTTACACGC-3’	390	NM_172890.3
GAT-3 (nested)	fwd 5’-GGGCATCTTCATCTTCTTTCTG-3′ rev 5’-AGTGTGTCTCCTTCTCTGTGAT-3’	320	
BGT-1	fwd 5’-ACTTTCTTCTTCTCCTTGAGCA-3′ rev 5’-CTCTGGCACTTCCTACAAATGA-3’	324	NM_133661.3
BGT-1 (nested)	fwd 5’-ACTTTCTTCTTCTCCTTGAGCA-3′ rev 5’-ATGAGTTCTTGTTTGGCTGGA-3’	284	
Kir4.1	fwd 5’-ACT TTC TTC TTC TCC TTG AGCA-3′ rev 5’-CTC TGG CAC TTC CTA CAA ATGA-3’	324	NM_001039484.1
Kir4.1 (nested)	fwd 5’-ACT TTC TTC TTC TCC TTG AGCA-3′ rev 5′-ATG AGT TCT TGT TTG GCT GGA-3’	284	

*fwd* Forward (/sence) primer, *rev* Reverse (/antisence) primer, *bp* Base pairs

Positive controls were performed with mRNA extracted from mouse brainstem by using an mRNA extraction kit (Dynabeads mRNA Purification Kit, Invitrogen; Additional file 3: Figure S3). For negative controls, the patch pipette was positioned close to the tissue in the recording chamber and ACSF was sucked into the pipette. Subsequently, the probe was frozen in liquid nitrogen and used for RT-PCR (Additional file 3: Figure S3). All amplified PCR products were loaded on an agarose gel (1.5%), labeled with 1% ethidium bromide (Carl Roth), and analyzed using a transilluminator (Biometra TI 1). To determine the PCR product length we used a standard DNA ladder (HyperLadder 50 bp, Bioline).

Initial experiments showed that some HC astrocytes were devoid of any target RNA (GlyTs or GATs). To prove successful RNA extraction from HC astrocytes transcripts for the inwardly rectifying K^+^ (Kir) channel 4.1 were detected, which are present in all HC astrocytes [46].

### Confocal microscopy

The labeling with SR101 - used for a priori identification of IC and HC astrocytes - and AF568 was documented as described before [41] using a confocal microscope (Leica TCS SP5 LSM: HC PL FLUOTAR 10 × 0.30 DRY; HCX PL APO Lambda blue 63 × 1.4 OIL UV) and LAS AF software. Fluorophores were detected as follows (excitation wavelength/filtered emission wavelength): SR101 (SP5: 561 nm/580-620 nm) and AF568 (561 nm/580-620 nm). To improve the quality of confocal micrographs and reduce background fluorescence, we used a Kalman filter (averaging of four identical image sections). Images were processed using Fiji software [47].

### Statistics

Results were statistically analyzed using WinSTAT (R. Fitch Software). Data were tested for normal distribution with Kolmogorov-Smirnov test. In case of normal distribution, results were assessed by one-tailed, paired or non-paired Student’s *t*-tests. In the absence of a normal distribution, results were assessed by Wilcoxon test for paired or U-test (Mann-Whitney) for non-paired data. *P* represents the error probability, *P < 0.05, **P < 0.01, ***P < 0.001; *n* represents the number of experiments or cells/slices/animals. In case of multiple comparisons data were statistically analyzed by the tests described above under post hoc Šidák correction of critical values [48]: two comparisons: Fig. 2a_3_, Fig. 4a_3_, Table 2; *P < 0.025, **P < 0.005, ***P < 0.0005; three comparisons: Fig. 2c_3_, Fig. 4c_3_, Additional file 2: Figure S2*B*_*3*_*-E*_*3*_, Table 3; *P < 0.017, **P < 0.0033, ***P < 0.0003. Data are provided as mean ± SEM.

**Table 2:** Glycine- and GABA-induced inward current and depolarization in IC and HC astrocytes

Region	*I*_Gly (max)_ [pA]	*P*	*I*_GABA (max)_ [pA]	*P*	Ratio	*P*	Δ*E*_M (Gly)_ [mV]	*P*	Δ*E*_M (GABA)_ [mV]	*P*	Ratio	*P*
IC	173 ± 28 (13)	0.200 n.s. sc	327 ± 35 (14)	0.009 * sc	1.9	0.002 ** sc	3.0 ± 0.8 (8)	0.374 n.s. sc	7.2 ± 0.8 (7)	0.041 n.s. sc	2.4	0.003 ** sc
HC	141 ± 20 (10)		504 ± 67 (7)		3.6	0.001 ** sc	2.6 ± 0.5 (6)		10.6 ± 1.6 (7)		4.1	0.002 ** sc

The data are given as mean ± SEM. The number of experiments is given in parenthesis. *IC* Inferior colliculus, *HC* Hippocampus, *P* Probability value, *sc* Šidák corrected significance level for 2 comparisons, *n.s.* Not significant, *sc: *P* < 0.025, **P < 0.005. For critical significance levels, see Methods section

**Table 3:** The effect of glycine and GABA on astrocytic and neuronal membrane resistance

Region	Cell type	Δ*R*_M (Gly)_ [%]	*P*	Δ*R*_M (GABA)_ [%]	*P*
IC	Astrocyte	+8.4 ± 3.6 (15)	0.018 n.s. sc	+1.1 ± 4.4 (15)	0.403 n.s. sc
	Neuron	-97.9 ± 0.5 (11)	0.000 ***sc	−98.9 ± 0.1 (11)	0.000 ***sc
HC	Astrocyte	+2.9 ± 4.0 (6)	0.252 n.s. sc	−7.6 ± 1.1 (8)	0.000 ***sc
	Neuron	−41.2 ± 6.2 (4)	0.004 *sc	−73.5 ± 5.7 (5)	0.000 ***sc

The data are given as mean ± SEM. The number of experiments is given in parenthesis. *IC* Inferior colliculus, *HC* Hippocampus, *P* Probability value, *sc* Šidák corrected significance level for 3 comparisons, *n.s*. Not significant, *sc: *P* < 0.017, ***sc: *P* < 0.0003. For critical significance levels, see Methods section. Δ*R*_M_ is given for t_1_ (1 s after focal application of neurotransmitter)

## Results

### Identification of IC and HC astrocytes

SR101 labeling is used in many different brain regions to identify astrocytes in acute tissue slices [19, 41, 42]. We mentioned before that incubation of acute slices with SR101 results in labeling of putative astrocytes in the IC, but we did not electrophysiologically confirm the identity of these SR101^+^ cells [19]. In IC and HC (CA1, *stratum radiatum*), SR101-labeled cells comprised a small sized soma with several branching processes. In HC, these cells exhibited strong branching, whereas in IC they appeared to be less complex (Fig. 1b, e). The SR101-labeled cells exhibited membrane properties of classical astrocytes, i.e. a highly negative membrane potential (*E*_M_: IC: -84.2 ± 0.3 mV, *n* = 207/116/101; HC: -81.8 ± 0.4 mV, *n* = 109/83/36) and a low *R*_M_ (IC: 8.3 ± 0.7 MΩ, *n* = 207/116/101; HC: 9.7 ± 0.6 MΩ, *n* = 109/83/36). Due to the presence of voltage-activated outward currents, non-passive and passive astrocytes were identified (IC: 28%/72%, *n* = 207/116/101, HC: 59%/41%, *n* = 109/83/36, Fig. 1c, f), which is typical for that developmental stage.

### GlyT expression in IC and HC astrocytes

Astrocytes in many brain regions express GlyTs [15], whereas GlyRs are only rarely present [20, 22]. To analyze the expression of functional GlyTs in IC and HC astrocytes, we first characterized the response of membrane current and potential upon glycine bath application. The wash-in caused a reversible glycine-induced inward current (*I*_Gly_) that usually peaked within the first minute (*I*_Gly (max)_: IC: 173 ± 28 pA, *n* = 13/11/10; HC: 141 ± 20 pA, *n* = 10/9/6; *P* = 0.200; Fig. 2a). Upon prolonged glycine administration, *I*_Gly_ partially recovered to a newly formed steady-state level in some recordings. Similarly, glycine induced a reversible depolarization (Δ*E*_M (Gly)_: IC: 3.0 ± 0.8 mV, *n* = 8/8/7; HC: 2.6 ± 0.5 mV, *n* = 6/6/5; *P* = 0.375; Fig. 2b). To prove whether *I*_Gly_ and Δ*E*_M (Gly)_ are mediated by GlyT1, we focally applied glycine in the absence and presence of sarcosine (Fig. 3a_1-2_). The competitive GlyT1 agonist itself caused an inward current by activation of the transporter and subsequently competed with applied glycine [19]. Sarcosine reduced *I*_Gly (max)_ by about 60-70% (IC: 59 ± 2%, *n* = 12/5/5, *P* < 0.001; HC: 70 ± 4%, *n* = 4/4/2, *P* = 0.015; Fig. 3b_3_) showing the presence of functional GlyT1 in IC and HC astrocytes.

As the inhibition of *I*_Gly (max)_ was incomplete and GlyT2 was occasionally reported to be present in astrocytes [40, 49], we analyzed transcripts for GlyTs in single astrocytes. GlyT1 mRNA was detected in all IC astrocytes and about half of the HC astrocytes (IC; n = 6/2/2; HC: *n* = 9/2/2; Fig. 3b). GlyT2 was never found in astrocytes but in the positive control (Additional file 3: Figure S3).

Interestingly, we never observed a glycine-induced outward current or changes in *R*_M_ (tested every 30 s ≙ 0.033 Hz) upon the activation of putatively expressed GlyRs (not shown). However, glycine-induced outward currents and *R*_M_ changes upon short term activation of GlyRs during the first seconds of glycine wash-in might be overlooked due to the relatively slow exchange of ACSF in the recording chamber and concomitant slow rise of the neurotransmitter concentration in combination with receptor desensitization [50]. Thus, bath application of glycine is not a suitable approach to prove the presence of functional GlyRs. Therefore, we designed a new protocol for fast and focal pressure injection of neurotransmitters in combination with a voltage-clamp protocol including a higher frequency of test pulses assessing *R*_M_ changes now at 1 Hz (Additional file 2: Figure S2*A*).

We first assessed the suitability of this protocol on IC and HC neurons. Bipolar shaped IC neurons and CA1 pyramidal cells (Additional file 1: Figure S1*A*) expressed time- and voltage-dependent inward and outward currents, respectively (Additional file 1: Figure S1*B*). Upon focal glycine application, IC neurons and CA1 pyramidal cells exhibited a transient, fast declining outward current (Additional file 2: Figure S2*B*_*1*_, *D*_*1*_). This was paralleled by an increase in the offset current induced by the test pulses resembling a strong reduction of *R*_M_ (IC: *t*_1_ − 97.9 ± 0.5%, *n* = 11/4/4, *P* < 0.001; HC: *t*_1_ -41.2 ± 6.2%, *n* = 4/2/2, *P* = 0.004; Additional file 2: Figure S2*B*_*2-3*_, *D*_*2-3*_). In the prolonged presence of glycine, *R*_M_ of IC neurons recovered partially, whereas *R*_M_ of CA1 pyramidal cells recovered completely (IC: *t*_10_: −90.9 ± 2.5% of resting *R*_M_, *P* < 0.001 compared to *t*_0_; *P* = 0.004 compared to *t*_1_; HC: *t*_10_: −4.5 ± 7.0% of resting *R*_M_, *P* = 0.285 compared to *t*_0_; *P* = 0.006 compared to *t*_1_). Both cases indicate desensitization of GlyRs (Additional file 2: Figure S2*B*_*3*_, *D*_*3*_), as previously reported for neurons in both regions [50, 51].

Subsequently, we used the focal application protocol on IC and HC astrocytes. Glycine induced an inward but no outward current at any time point during the 10 s application (Fig. 3c_1-2_). Furthermore, the offset current induced by the test pulses did not change. At *t*_1_ (1 s after glycine application), *R*_M_ was not reduced (IC: +8.4 ± 3.6%, *n* = 15/4/4, *P* = 0.018; HC: +2.9 ± 4.0%, *n* = 6/5/3, *P* = 0.252; Fig. 3c_3_). Thus, *R*_M_ was glycine-independent arguing against an activation of GlyRs. Taken together, IC and HC astrocytes expressed functional GlyT1, whereas GlyRs were only present in IC and HC neurons. Data are summarized in Tables 2 and 3.

### GAT expression in IC and HC astrocytes

GATs are present in astrocytes of various brain regions [15, 16]. Here, we analyzed the expression of different functional GATs in IC and HC astrocytes. GATs and GABA_A_Rs mediate – under our experimental conditions – an inward and outward current, respectively. The wash-in of GABA induced a transient inward current (*I*_GABA_) that peaked usually within the first minute (*I*_GABA (max)_; Fig. 4a_1-2_). Notably, *I*_GABA (max)_ was larger in HC astrocytes (IC: 327 ± 35 pA, *n* = 14/11/10; HC: 504 ± 67 pA, *n* = 7/7/5; *P* = 0.009; Fig. 4a_3_). Upon prolonged application, *I*_GABA_ recovered occasionally to a lower steady-state level in some recordings. Similar to *I*_GABA_, GABA induced a reversible depolarization (IC: Δ*E*_M (GABA)_: 7.2 ± 0.8 mV, *n* = 7/7/7; HC: Δ*E*_M (GABA)_: 10.6 ± 1.6 mV, *n* = 7/7/5; *P* = 0.041; Fig. 4b). Both *I*_GABA_ and Δ*E*_M (GABA)_ indicate the presence of functional GATs. Noticeably, GABA-induced transients were 2-4-fold larger than the above-described glycine-induced transients. In addition, this difference was more prominent in HC astrocytes than in IC astrocytes (Table 2).

To assess the different GAT isoforms being expressed in IC and HC astrocytes, we focally applied GABA and analyzed the sensitivity of *I*_GABA_ to the non-competitive GAT-1 and GAT-3 antagonists NO711 and SNAP5114, respectively (Fig. 5a_1-2_). The two antagonists reduced *I*_GABA (max)_ by about 20-40% (IC: NO711: 19 ± 4%, *n* = 4/4/2, *P* = 0.007; SNAP5114: 22 ± 4%, *n* = 4/4/4, *P* = 0.003; HC: NO711: 28 ± 6%, *n* = 8/8/5, *P* < 0.001; SNAP5114: 43 ± 6%, *n* = 7/7/4, *P* = 0.001; Fig. 5a_3_) showing the presence of functional GAT-1 and GAT-3. NO711 and SNAP5114 themselves had no effect on the membrane current. Simultaneously inhibiting GAT-1 and GAT-3 led to an incomplete reduction of *I*_GABA (max)_ (Fig. 5a_1-2_). This can either result from a low antagonist concentration that was chosen in order to ensure specificity of the substances or from the presence of further GATs, i.e. GAT-2 (*Slc6A13*) and BGT-1 (*Slc6A12*) [16]. The latter case was addressed analyzing transcripts for the four cloned GATs in single astrocytes. All tested IC astrocytes exhibited transcripts for GAT-1 and GAT-3, whereas these transporters are present in about half of the HC astrocytes. It has to be pointed out that transcripts for GAT-1 and GAT-3 were found in 3/7 HC astrocytes, whereas they were not detected in 3/7 cases. In those cells transcripts for Kir4.1 were found, which proved successful RNA extraction. One HC astrocyte expressed only transcripts for GAT-1. However, transcripts for GAT-2 and BGT-1 were only detected in the positive control (Additional file 3: Figure S3), but not in individual IC or HC astrocytes (IC: *n* = 3/2/2; HC: n = 7/6/5; Fig. 5b).

Astrocytes, for example in the HC, express GABA_A_Rs [21, 22]. Accordingly, *I*_GABA (max)_ might be underestimated if GABA_A_R-mediated Cl^−^ influx causes an outward current that counteracts the GAT-mediated inward current. To verify this, we performed fast and focal pressure injection of GABA and assessed *R*_*M*_ changes.

Positive controls on GABA_A_R expressing IC and HC neurons [30, 50, 52] showed the suitability of the experimental configuration to reveal GABA_A_R activation. Focal GABA application induced a transient, fast declining outward current (Additional file 2: Figure S2*C*_*1*_, *E*_*1*_). This was paralleled by strong increase in the offset current induced by the test pulses resembling an eminent reduction of *R*_M_ (IC: *t*_1_: −98.9 ± 0.1%, *n* = 11/6/6, *P* < 0.001; HC: *t*_1_: −73.5 ± 5.7%, *n* = 5/3/2, *P* < 0.001; Additional file 2: Figure S2*C*_*2-3*_, Additional file 2: Figure S2*E*_*2-3*_). In the prolonged presence of GABA, *R*_M_ of IC neurons recovered partially indicating minimal desensitization of GABA_A_Rs (*t*_10_: −96.4 ± 0.4% of resting *R*_M_, *P* < 0.001 compared to *t*_0_; *P* < 0.001 compared to *t*_1_; Additional file 2: Figure S2*C*_*3*_) as previously reported [50]. In contrast, *R*_M_ of CA1 pyramidal cells recovered completely indicating strong desensitization of GABA_A_Rs (*t*_10_: +2.8 ± 9.4% of resting *R*_M_, *P* = 0.250 compared to *t*_0_; *P* = 0.003 compared to *t*_1_; Additional file 2: Figure S2*E*_*3*_) as previously reported [53, 54].

Subsequently, we did focal application and analyzed putative GABA_A_R-mediated *R*_M_ changes in IC and HC astrocytes (Fig. 5c). At any time, GABA induced an inward but no outward current (Fig. 5c_1-2_). In IC astrocytes, the offset current induced by the test pulses did not change (Fig. 5c_1_). Accordingly, at *t*_1_*R*_M_ was not reduced (+1.1 ± 4.4%, *n* = 15/5/5, *P* = 0.403; Fig. 5c_3_). Thus, *R*_M_ was GABA-independent arguing against an activation of GABA_A_Rs. In HC astrocytes however, GABA increased the offset current in response to the test pulses (Fig. 5*c*_*2*_). In turn, *R*_M_ was reduced (*t*_1_: −7.6 ± 1.1%, *n* = 8/5/2, *P* < 0.001; Fig. 4c_3_) demonstrating activation of GABA_A_Rs in HC astrocytes. In the prolonged presence of GABA, *R*_M_ recovered completely, indicating desensitization of GABA_A_Rs (*t*_10_: −2.4 ± 1.3% of resting *R*_M_, *P* = 0.055 compared to *t*_0_; *P* < 0.001 compared to *t*_1_; Fig. 5c_3_). Thus, *I*_GABA (max)_ was not contaminated by GABA_A_R activation as it was not determined within the first 10 s of GABA wash-in. Taken together, IC and HC astrocytes co-expressed functional GAT-1 and GAT-3, whereas GABA_A_Rs were only found in HC astrocytes. Data are summarized in Tables 2 and 3.

### Region-dependent transporter kinetics

**Fig. 6:**
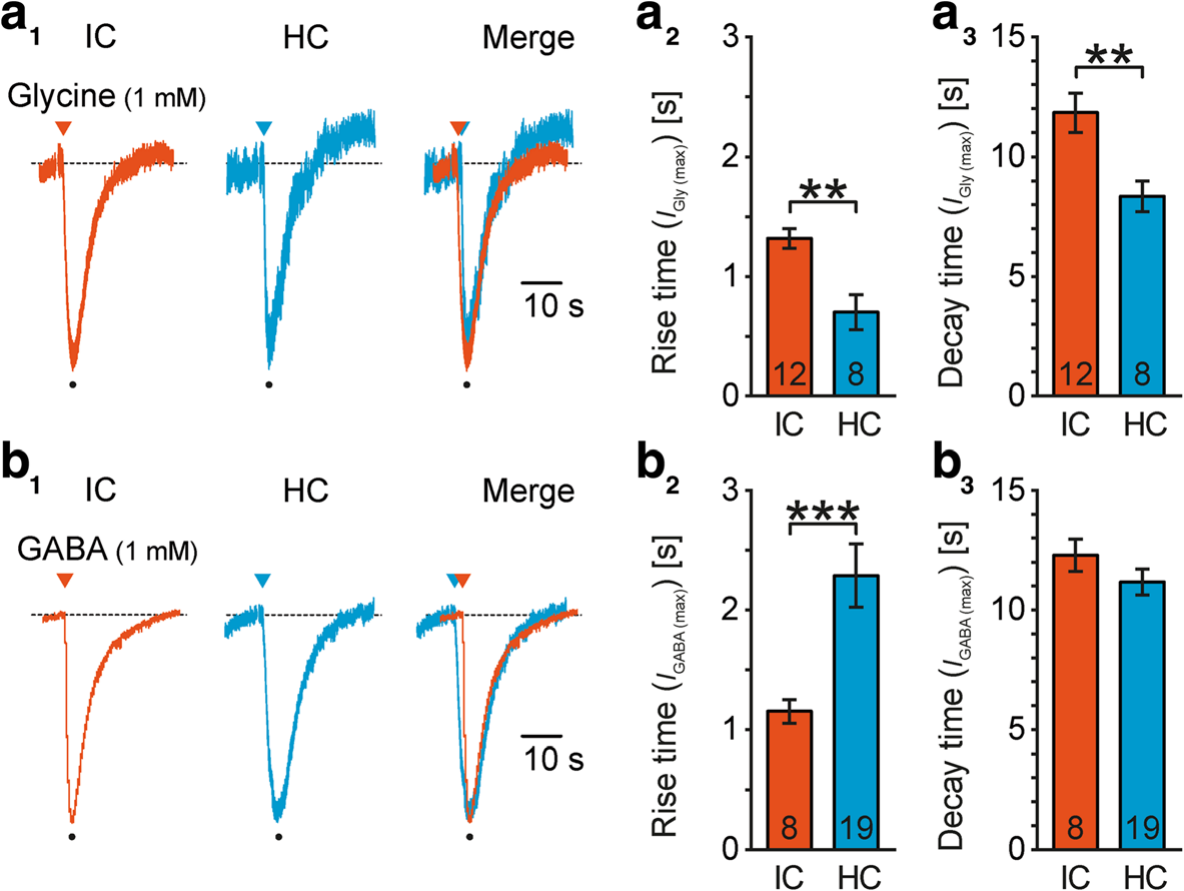
Region-dependent kinetics of *I*_Gly_ and *I*_GABA_. **a:** *I*_Gly_ kinetics. Focal application of glycine (1 mM, 0.5 s; triangles) elicited an *I*_Gly_ (*E*_H_ = −85 mV) in IC (left) and HC (middle) astrocytes. Merged traces were peak aligned (right; a_1_). IC astrocytes exhibited longer rise time (a_2_; 10-90) and decay time (a_3_; 90-10) compared to HC astrocytes. **b:** *I*_GABA_ kinetics. Focal application of GABA (1 mM, 0.5 s; triangles) elicited an *I*_GABA_ (*E*_H_ = −85 mV) in IC (left) and HC (middle) astrocytes. Merged traces were peak aligned (right; b_1_). IC astrocytes exhibited a shorter rise time compared to HC astrocytes (b_2_). The decay time was not significantly different (b_3_). The number of recorded cells (*n*) is given within the diagrams. Shown are mean values ± SEM

As we observed that the *I*_GABA (max)_/*I*_Gly (max)_ ratio was larger in HC compared to IC (Table 2) we speculated about putative additional differences between those nuclei regarding transporters kinetics. Thus, we analyzed rise time (10 - 90%) and decay time (90 - 10%) of *I*_Gly_ and *I*_GABA_ in IC and HC astrocytes resulting from focal application of glycine and GABA (Fig. 6a_1_, b_1_). The rise time of *I*_Gly_ was much shorter in HC astrocytes (IC: 1.32 ± 0.08 s, *n* = 12/5/5; HC: 0.70 ± 0.15 s, *n* = 8/7/4; *P* = 0.002; Fig. 6a_2_). Additionally, the decay time of *I*_Gly_ was shorter in HC astrocytes, too (IC: 11.83 ± 0.83 s, *n* = 12/5/5; HC: 8.35 ± 0.64 s, *n* = 8/7/4; *P* = 0.002; Fig. 6a_3_). Together, HC astrocytes exhibited faster kinetics for *I*_Gly_.

Similarly, we analyzed the kinetics of *I*_GABA_ (Fig. 6b). Here, IC astrocytes exhibited a much shorter rise time (IC: 1.15 ± 0.10 s, *n* = 8/8/8; HC: 2.29 ± 0.27 s, *n* = 19/19/6; *P* < 0.001; Fig. 6b_2_). The decay time of *I*_GABA_ was not different between IC and HC astrocytes (IC: 12.29 ± 0.68 s, *n* = 8/8/8; HC: 11.17 ± 0.54 s, *n* = 19/19/6; *P* = 0.123; Fig. 6b_3_). Taken together, our data demonstrate that transporter-mediated currents were heterogeneous with respect to glycine and GABA as well as the brain region. HC astrocytes exhibited faster *I*_Gly_ kinetics, whereas IC astrocytes partially showed faster *I*_GABA_ kinetics. Data are summarized in Table 4.

**Table 4:** Kinetics of glycine- and GABA-induced currents in IC and HC astrocytes

Region	*I*_Gly_				*I*_GABA_			
	Rise time [s]	*P*	Decay time [s]	*P*	Rise time [s]	*P*	Decay time (s]	*P*
IC	1.32 ± 0.08 (12)	0.002**	11.83 ± 0.83 (12)	0.002**	1.15 ± 0.10 (8)	0.000***	12.29 ± 0.68 (8)	0.123 n.s
HC	0.70 ± 0.15 (8)		8.35 ± 0.64 (8)		2.29 ± 0.27 (19)		11.17 ± 0.54 (19)	

The data are given as mean ± SEM. The number of experiments is given in parenthesis. *IC* Inferior colliculus, *HC* Hippocampus, *P* Probability value, *n.s*. Not significant

**: *P* < 0.01, ***: *P* < 0.001

In summary, our results show that SR101-labeled cells in the IC and HC exhibited properties of classical astrocytes. In all IC and about half of the HC astrocytes, GlyT1, GAT-1, and GAT-3 were present, whereas GlyT2, GAT-2, and BGT-1 were not found. In both regions, astrocytes exhibited a stronger GAT than GlyT activity. However, in HC astrocytes the *I*_GABA (max)_/*I*_Gly (max)_ ratio was remarkably higher. In comparison to IC astrocytes HC astrocytes showed faster kinetics for the transport of glycine and slower kinetics for the transport of GABA. Finally, GlyRs could not be detected in astrocytes of IC and HC. However, expression of GABA_A_Rs was heterogeneous – it was found in HC but not in IC astrocytes.

## Discussion

In the present study, we investigated the expression and function of GlyTs and GATs in astrocytes from IC and HC. In both regions, astrocytes generally expressed the three inhibitory neurotransmitter transporters GlyT1, GAT-1 and GAT-3, whereas GlyT2, GAT-2, and BGT-1 were not detected. Remarkably, IC astrocytes exhibited larger *I*_Gly (max)_ and smaller *I*_GABA (max)_ compared to HC astrocytes. In turn, this resulted in a higher *I*_GABA (max)_/*I*_Gly (max)_ ratio in HC astrocytes.

### Basic properties of IC and HC astrocytes

Astrocytes were labeled with SR101, by which classical astrocytes in acute tissue slices – containing the superior olivary complex (SOC) or the HC – can be identified [19, 41, 42, 55]. We mentioned before that in the IC SR101 labels small sized and highly branched cells [19]. However, their identity was not verified yet by electrophysiological recordings. Here we show that SR101-labeled IC cells exhibit a highly negative *E*_M_ and a low *R*_M_. They are not NG2 glia as these exhibit completely different electrophysiological properties, i.e. a more positive *E*_M_, a tremendously higher *R*_M_, and currents through voltage-activated sodium channels [42, 56–58]. Furthermore, they are unlikely to be oligodendrocytes as they are if at all just weakly labeled by SR101 [41]. In contrast, SR101-labeled IC cells exhibited a non-linear or linear current-voltage relationship corresponding to non-passive and passive astrocytes, respectively, which are found throughout the auditory brainstem (Fig. 1) [19, 41, 55, 59, 60]. Furthermore, these from now on IC astrocytes-termed cells were distributed homogeneously within the nucleus (Fig. 1) like astrocytes in SOC nuclei [19, 41]. HC astrocytes exhibited properties like reported in previous studies, e.g. [42, 56].

### IC and HC astrocytes express functional GlyT1, GAT-1, and GAT-3

Glycine and GABA activate respective transporters that mediate an inward current and a concomitant depolarization due to their stoichiometry: 1 glycine/1 GABA: 2 Na^+^: 1 Cl^−^ [15, 17]. Both inward current and depolarization sometimes partially recovered in the prolonged presence of the agonist (Figs. 2 and 4). This was observed before in LSO astrocytes and may be due to a reduced driving force [19]. Both IC and HC astrocytes showed sarcosine-sensitive *I*_Gly (max)_, demonstrating the presence of functional GlyT1 (Fig. 3). Sarcosine is a competitive agonist and therefore inhibited just about 60-70% of *I*_Gly (max)_ [61]. Thus, the co-expression of the neuron-typical GlyT2 could not be excluded per se. GlyT2 was reported to be present occasionally in astrocytes [40, 49]. However, here we never found transcripts for GlyT2 in IC and HC astrocytes indicating the absence of GlyT2. GlyT1 mRNA was present in all IC astrocytes sufficiently explaining *I*_Gly_. However, GlyT1 transcripts were found only in about half of the HC astrocytes (Fig. 3). There are several possible explanations: 1) Although the scRT-PCR reliably detected GlyT1 transcripts in the positive controls, it was eventually not sensitive enough to detect single transcripts in all HC astrocytes. 2) There is effectively a mosaic expression of GlyT1. However, all recorded HC astrocytes exhibited an *I*_Gly_ (Fig. 2). Thus, HC astrocytes putatively express further transporters that are capable to transport glycine. The neutral amino acid transporter ASCT2 (*Slc1A5*) as well as sodium-coupled neutral amino acid transporters (system N) SNAT3 (*Slc38A3*) and SNAT5 (*Slc38A5*) are expressed by astrocytes and transport glycine, but are electroneutral and accordingly do not generate currents [62–65]. 3) HC astrocytes are extensively coupled [66–69] and allow direct electrical communication between neighboring astrocytes [70–72]. Here, about half of the HC astrocytes lacked GlyT1 expression, but can be expected to be surrounded by and coupled to GlyT1 expressing HC astrocytes. Therefore, GlyT1 negative astrocytes might indirectly experience *I*_Gly_.

Likewise, IC and HC astrocytes exhibited NO711- and SNAP5114-sensitive *I*_GABA (max)_ showing the co-expression of functional GAT-1 and GAT-3 in both regions (Fig. 5). Hitherto, in the HC GAT-1 and GAT-3 were attributed to interneurons and astrocytes, respectively [21, 37]. To our surprise, we found prominent expression of functional GAT-1 in HC astrocytes. NO711 and SNAP5114 inhibited *I*_GABA (max)_ by about 20 to 40% (Fig. 5), which is similar to our former study on LSO astrocytes [19]. However, simultaneous administration of NO711 and SNAP5114 did not completely abolish *I*_GABA (max)_ (Fig. 5). Both antagonists dose-dependently inhibit respective GATs [21]. As we here used a low drug concentration to retain the specificity of GAT inhibitors [16] it was not expected to achieve a complete blockage. However, to that point our data did not exclude the possibility of co-expression of further GATs, such as GAT-2 or BGT-1. The latter are predominantly found at the meninges and neuronal somata, respectively [16]. In accordance, we found only transcripts for GAT-1 and GAT-3 but not for GAT-2 and BGT-1 in IC and HC astrocytes. These results indicate that *I*_GABA_ was solely mediated by GAT-1 and GAT-3 (Fig. 5). Surprisingly, GAT-1 and GAT-3 mRNA exhibited a mosaic pattern in HC astrocytes. In 3/7 cases HC astrocytes did not exhibit transcripts for any GAT. There are two possible explanations: 1) Although the scRT-PCR detected transcripts in the positive controls, it was not sensitive enough to detect single transcripts on the single cell level. 2) There is effectively a mosaic expression pattern. However, the second explanation contrasts with the finding that all HC astrocytes exhibited *I*_GABA_ that was always sensitive to the GAT-1 and GAT-3 inhibitor NO711 and SNAP5114, respectively (Figs. 4 and 5). Again, the extensive coupling of and direct electrical communication between HC astrocytes [66–72] could explain why *I*_GABA_ was recorded in all cells independent from GAT expression.

The co-expression of GlyTs and GATs in the same astrocyte raises the question of transporter interference. Such interference of different transporters was seen before [6, 73, 74]. In a previous study on LSO astrocytes, we could show that GlyT and GAT activity influence each other [19]. The reciprocal reduction of activity likely refers to changes in their commonly used gradients for Na^+^ and Cl^−^. Those gradients become weakened upon transporter activation thereby reducing the driving force for the transport. Especially in the IC, where neurons simultaneously receive glycinergic and GABAergic synaptic inputs [29, 30], transporter interference might occur during synchronous activation of astrocytic GlyTs and GATs [15]. However, it remains to be elucidated to which extend this interplay takes place and how altered neurotransmitter clearance putatively modulates neuronal signaling [15, 17, 18].

Taken together, all IC and about half of the HC astrocytes expressed functional GlyT1, GAT-1, and GAT-3. In this respect, these astrocytes can express the same combination of inhibitory neurotransmitter transporters like astrocytes located in LSO, thalamus, cortex or Bergmann glia in the cerebellum or Müller cells in the retina [15, 19, 75–80]. The potentially heterogeneous expression in HC astrocytes could be indicative of functional domains in which glycinergic transmission arising from excitatory projections and GABAergic transmission from interneurons are segregated from each other.

### Transporter currents are not contaminated by ionotropic receptor activation

Both glycine and GABA act on respective transporters and ionotropic receptors. While activation of GlyTs and GATs by exogenous accessible neurotransmitters necessarily causes an inward current, activation of GlyRs and GABA_A_Rs can result in either an inward current or an outward current. The underlying Cl^−^ efflux or influx depends on [Cl^−^]_i_ and subsequently on *E*_Cl_. Under physiological conditions astrocytic [Cl^−^]_i_ amounts to about 30 mM [81] causing an inward current and concomitant depolarization upon receptor activation. However, our pipette solution contained 2 mM Cl^−^ and receptor activation would have caused an outward current. In our recordings, we never observed glycine- or GABA-induced outward currents in IC and HC astrocytes (e.g. Figs. 2 and 4), which was surprising as at least HC astrocytes express functional GABA_A_Rs [21, 22]. Two possible scenarios could explain this discrepancy: 1) The GABA_A_R-mediated outward current was too small and consecutively masked by the large GAT-mediated inward current. This in turn would suggest that the amount of GAT-mediated inward current would be underestimated. 2) GABA_A_Rs rapidly desensitize [53, 54, 82]. In combination with slow wash-in of GABA in our experiments, this early desensitization might hamper the accurate detection of GABA_A_R activation. To answer the question of masked activation and/or desensitization of ionotropic receptors we measured *R*_M_ changes that could result from increased membrane permeability (see Methods). Proof of principle experiments on GlyR and GABA_A_R expressing IC and HC neurons validated the method (Additional file 2: Figure S2). Our results convincingly demonstrated the capability to detect *R*_M_ changes upon GlyR and GABA_A_R activation with the utilized test pulse protocol.

With this tool at hand, we were able to detect GABA_A_R activation in HC astrocytes (Fig. 5). GABA_A_R activation was detected by temporary *R*_M_ reduction that vanished within 10 s indicating receptor desensitization. However, we never observed any outward current that had to arise from Cl^−^ influx due to the low [Cl^−^]_i_ of the intracellular solution. We reason that any small Cl^−^ influx-mediated outward current is instantly masked by strong electrogenic transporter current. Nonetheless, *I*_GABA (max)_, which was measured earliest after 10 s, was not contaminated by GABA_A_R-mediated currents. The *R*_M_ reduction in HC astrocytes was rather small (~8%) compared to HC neurons (~74%). Astrocytes express various K^+^ channels that are constitutively open at resting conditions (inwardly rectifying K^+^ channels, two-pore-domain K^+^ channels) [46, 83]. In turn, these channels cause the very high K^+^ conductance observed in astrocytes [44]. Accordingly, it is not surprising that the *R*_M_ reduction was relatively small. At the same time, IC astrocytes exhibited no *R*_M_ reduction upon GABA application (Fig. 5). Thus, either GABA_A_Rs are absent or their amount is essentially not high enough to be relevant. Interestingly, using this method on LSO astrocytes we detected a small *R*_M_ reduction indicating the presence of GABA_A_Rs (Vanessa Augustin and Simon Wadle, unpublished). We previously reported that *I*_GABA_ in LSO astrocytes mainly constitutes of GAT-mediated current [19]. Similar to HC astrocytes, the GABA-induced *R*_M_ reduction in LSO astrocytes vanished within 10 s after the beginning of GABA application. Thus, our previously reported *I*_GABA (max)_ in LSO astrocytes was not contaminated by GABA_A_R activation.

Similarly, we used the same method to examine a possible influence of GlyR activation onto our recorded *I*_Gly (max)_. We could show that neither IC nor HC astrocytes exhibited glycine-induced *R*_M_ changes or outward currents (Figs. 2 and 3). Likewise, LSO astrocytes lack glycine-induced *R*_M_ changes (Vanessa Augustin and Simon Wadle, unpublished). Accordingly, functional GlyRs appear to be absent in those astrocytes. This is consistent with the observation that GlyRs were described only in astrocytes located in most caudal brain regions, i.e. spinal cord and caudal brainstem (ventral respiratory group) [20, 24, 25]. However, this contrasts with the wide distribution of GABA_A_Rs throughout the brain [22]. In summary, *I*_Gly (max)_ and *I*_GABA (max)_ were not affected by GlyRs and GABA_A_Rs, respectively, and the transporter currents were accordingly not underestimated.

### Activity and kinetics of GlyTs and GATs

IC and HC astrocytes are differently capable to take up glycine and GABA (Table 2). While there is no statistical difference for glycine transport among the two brain regions, the GABA transport is stronger in HC astrocytes. In the LSO, which is located more caudal compared to IC and HC, astrocytes exhibit a similar capability to take up glycine. However, their ability for GABA clearance is much lower [19]. Thus, astrocytic *I*_GABA (max)_ increases from caudal to rostral brain regions (LSO < IC < HC). Consequently, the ratio of *I*_GABA (max)_/*I*_Gly (max)_ is elevated in more rostral brain regions (HC (3.6) > IC (1.9) > LSO (1.6; data from [19])). This was expected, as the need to take up GABA rather than glycine is higher in rostral brain regions, which arises from the glycine-to-GABA shift as the predominant inhibitory neurotransmitter [2, 3, 9, 10, 29, 30]. Noticeably, GlyT-mediated *I*_Gly (max)_ substantially persists in HC astrocytes. This allows the clearance of glycine that is co-released from excitatory presynaptic terminals [11, 12]. Taken together, *I*_Gly (max)_ is similar in the three brain regions, whereas *I*_GABA (max)_ as well as the *I*_GABA (max)_/*I*_Gly (max)_ ratio are region-dependent and increases with the prevalence of GABA as inhibitory neurotransmitter.

Beside inter-region differences of amplitudes, we additionally found region-dependent alterations of the kinetics of transporter-mediated currents (Table 4). Whereas IC astrocytes exhibit similar kinetics for the transport of glycine and GABA, HC astrocytes are marked by faster glycine and slower GABA transport. However, LSO astrocytes generally outperform IC and HC astrocytes regarding kinetics of GlyTs (rise time: 1.05 ± 0.18 s; decay time: 4.88 ± 1.11 s; *n* = 6/6/6) and GATs (rise time: 0.61 ± 0.13 s; decay time: 4.52 ± 0.52 s; *n* = 12/12/11; data from [19]). GlyTs and GATs can be modulated by several mechanisms: e.g., enhancement of transporter activity can be achieved by transporter glycosylation and [Ca^2+^]_i_ elevation [84–86], whereas decrease of transporter activity can be caused by activation of protein kinase C and de-glycosylation [84, 85, 87–89]. If one or more of those mechanisms are relevant in astrocytes of the three brain regions, is yet unexplored. However, the different transport kinetics correlate with different precision of signal processing in those three brain regions. The auditory system in general requires temporal precise coding to compute correctly e.g. interaural time and level differences in the medial superior olive and the LSO, respectively, and synapses show relatively weak depression allowing high rates of synaptic transmission [4, 90–92]. Furthermore, the synaptic signaling in the LSO is considerably faster and more precise compared to the hippocampus [93]. Like the LSO, the IC belongs to the auditory brainstem. However, it is not used for sound source localization, but serves as an information hub. Thus, the IC can tolerate a slower and less precise synaptic transmission. As the rate of neurotransmitter transporter activity determines the extent of synaptic transmission [17, 18], the fast transmitter uptake into LSO astrocytes to terminate quick synaptic transmission is in favor of fast and precise neuronal signaling. In contrast, synaptic transmission in IC and HC is not as precise and neurotransmitter uptake is not that fast. Thus, our data suggest that expression and kinetics of astrocytic inhibitory neurotransmitter transporters are adjusted to the requirements of local circuitry.

## Conclusion

In summary, our results demonstrate the expression of functional GlyT1, GAT-1, and GAT-3 in all IC astrocytes and about half of the HC astrocytes. In both regions the activity of GATs is stronger compared to the activity of GlyTs. Whereas *I*_Gly (max)_ is comparable in both regions, *I*_GABA (max)_ is much larger in HC astrocytes. Accordingly, the *I*_GABA (max)_/*I*_Gly (max)_ ratio is markedly elevated in HC astrocytes. Furthermore, astrocytic GlyTs and GATs in IC as well as HC exhibit slower transporter kinetics in comparison to those transporters in LSO astrocytes, thereby reflecting the regionally differing demands for temporal precision of synaptic transmission. Altogether, our results show that astrocytes do not uniformly express inhibitory neurotransmitter transporters, but region specifically adapt to the requirements of local circuitry.

## Additional files

Additional file 1: Figure S1. Basic characterization of IC and HC neurons. A: Reconstruction of a single IC and HC neuron. Dendrite topography of the IC neuron correlated with isofrequency bands (dorsomedial to ventrolateral orientation; *A*_*1*_). Basal and apical dendrites from CA1 pyramidal cell extended into *stratum oriens* (*SO*) and *stratum radiatum* (*SR*), respectively (*A*_*2*_). B: Neurons were clamped to *E*_H_ = −70 mV and were stepwise hyper- and depolarized from −150 mV to +50 mV, with 10 mV increments. IC (*B*_*1*_) and HC (*B*_*2*_) neurons expressed voltage-dependent early inward and delayed outward currents. Inset: higher temporal resolution of inward currents. Scale bars: 1 ms. (TIFF 1876 kb)

Additional file 2: Figure S2. Glycine and GABA induced *R*_M_ reduction in IC and HC neurons. A: Voltage-clamp protocol including 11 test pulses (*t*_0-10_). B-E: Neurons were clamped to *E*_H_ = −70 mV. Focal application of glycine or GABA (1 mM, 10 s; triangles) induced a transient outward current (*B*_*1*_*-E*_*1*_). Responses to test pulses at *t*_0_, *t*_1_, *t*_5_, and *t*_10_ (*B*_*2*_*-E*_*2*_) allowed calculation of *R*_M_. Upon glycine or GABA application *R*_M_ was reduced in IC and HC neurons (*B*_*3*_*-E*_*3*_). *n* represents the number of recorded cells. Shown are mean values ± SEM. Significance levels in panels *B*_*3*_*-E*_*3*_ were Šidák corrected for three comparisons (see Methods section). (TIFF 2112 kb)

Additional file 3: Figure S3. Controls for single-cell RT-PCR. Random RNA prepared from brainstem served as positive control (p.c.). For negative control (n.c.) a patch pipette was dipped into ACSF and was placed closely to the surface of the slice without patching a cell. All targeted mRNAs were detected in p.c., whereas the n.c. was free of signals. (TIFF 1434 kb)

## Abbreviations

AF: Alexa fluor
*E*_H_: Holding potential
GABA_A_R: GABA_A_ receptor
GAT: GABA transporter
GlyT: Glycine transporter
HC: Hippocampus
IC: Inferior colliculus
*I*_GABA (max)_: Maximal GABA-induced current
*I*_GABA_: GABA-induced current
*I*_Gly (max)_: Maximal glycine-induced current
*I*_Gly_: Glycine-induced current
LSO: Lateral superior olive
NMDAR: NMDA receptor
*R*_M_: Membrane resistance
*R*_S_: Series resistance
RT-PCR: Reverse transcription PCR
SOC: Superior olivary complex
