# Supplementary figures and images for: Expression of functional inhibitory neurotransmitter transporters GlyT1, GAT-1, and GAT-3 by astrocytes of inferior colliculus and hippocampus
Source: Mol Brain. 2018 Jan 25;11:4. doi: 10.1186/s13041-018-0346-y (PMC5785846; doi:10.1186/s13041-018-0346-y)

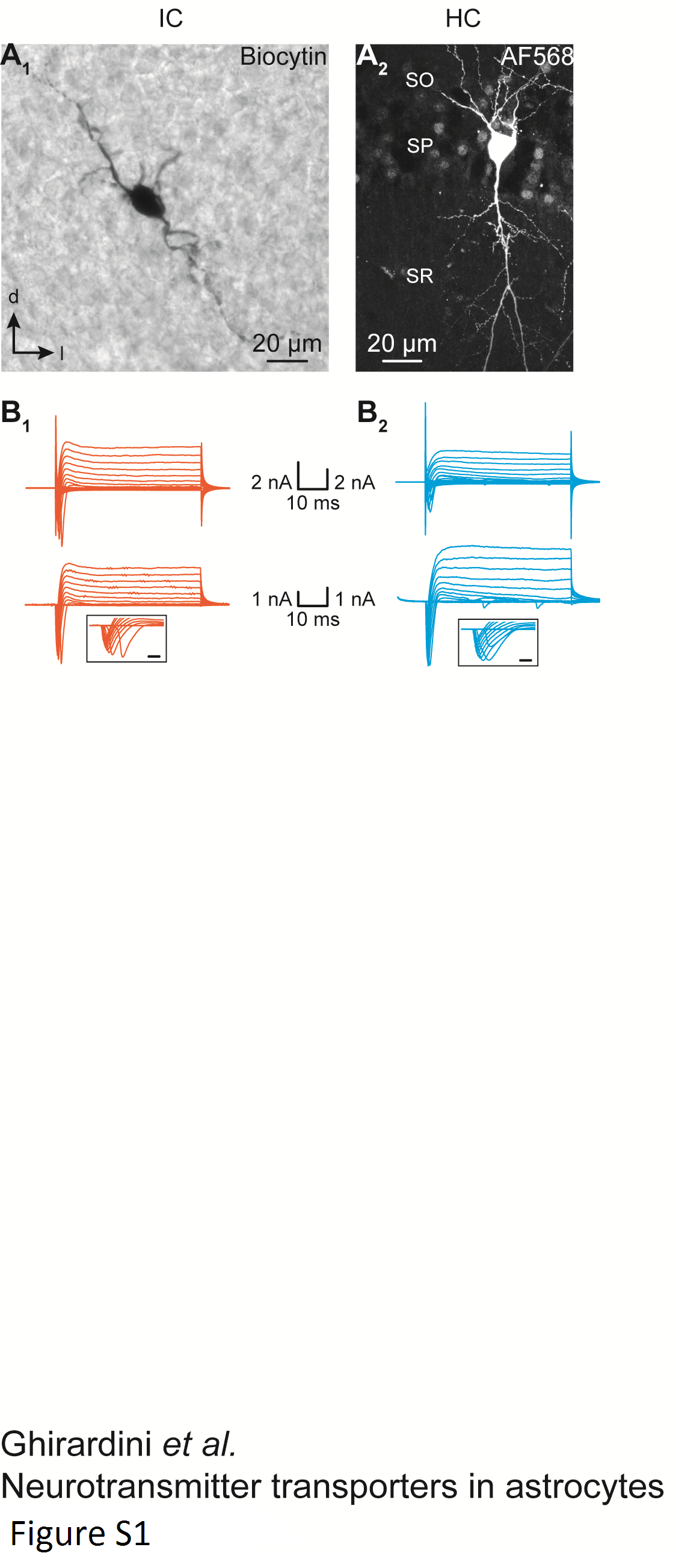

Supplement: Supplementary file 1 — Basic characterization of IC and HC neurons. A: Reconstruction of a single IC and HC neuron. Dendrite topography of the IC neuron correlated with isofrequency bands (dorsomedial to ventrolateral orientation; A1). Basal and apical dendrites from CA1 pyramidal cell extended into stratum oriens (SO) and stratum radiatum (SR), respectively (A2). B: Neurons were clamped to EH = −70 mV and were stepwise hyper- and depolarized from −150 mV to +50 mV, with 10 mV increments. IC (B1) and HC (B2) neurons expressed voltage-dependent early inward and delayed outward currents. Inset: higher temporal resolution of inward currents. Scale bars: 1 ms. (TIFF 1876 kb) [file 13041_2018_346_MOESM1_ESM.tif]

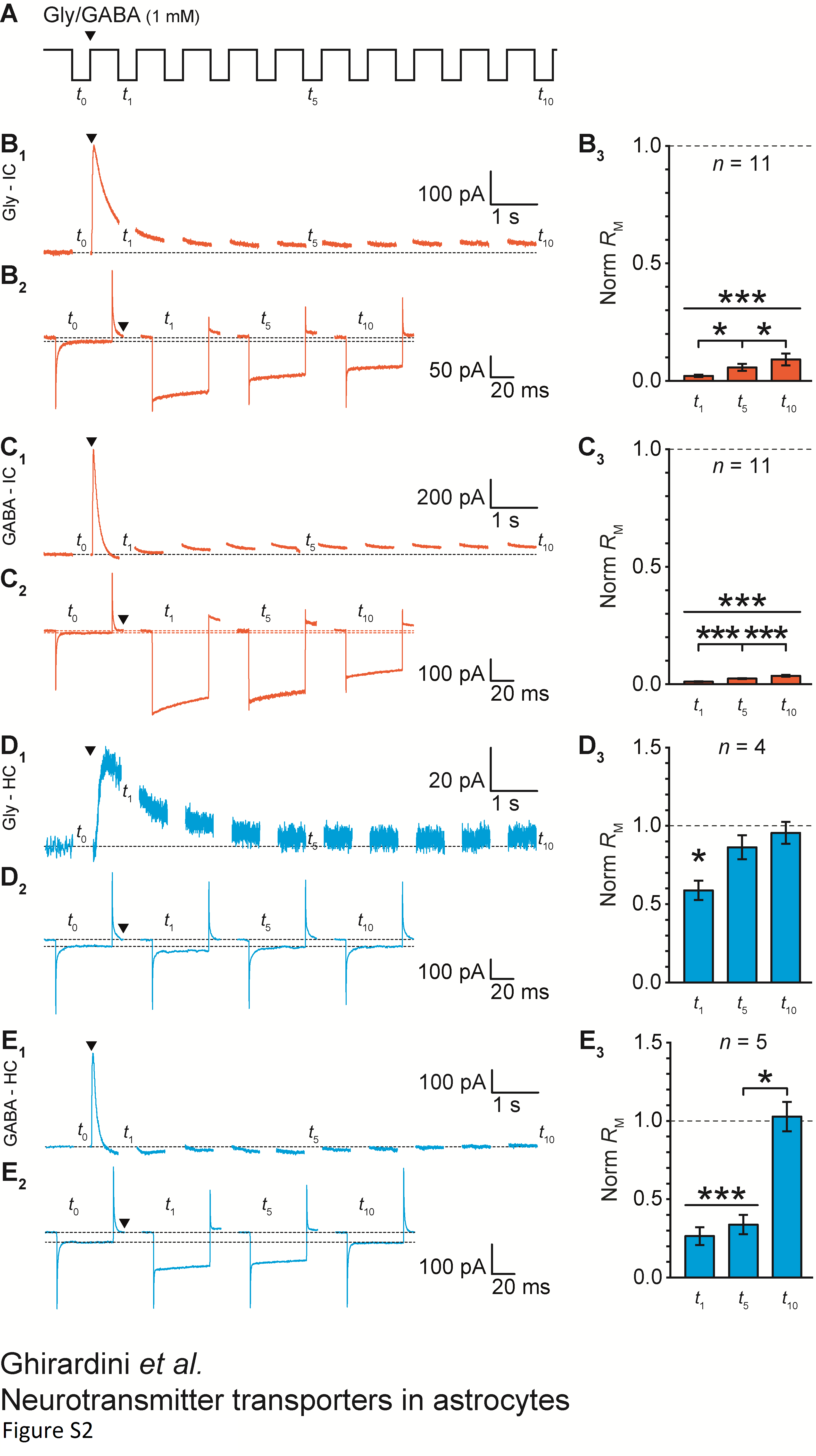

Supplement: Supplementary file 2 — Glycine and GABA induced RM reduction in IC and HC neurons. A: Voltage-clamp protocol including 11 test pulses (t0-10). B-E: Neurons were clamped to EH = −70 mV. Focal application of glycine or GABA (1 mM, 10 s; triangles) induced a transient outward current (B1-E1). Responses to test pulses at t0, t1, t5, and t10 (B2-E2) allowed calculation of RM. Upon glycine or GABA application RM was reduced in IC and HC neurons (B3-E3). n represents the number of recorded cells. Shown are mean values ± SEM. Significance levels in panels B3-E3 were Šidák corrected for three comparisons (see Methods section). (TIFF 2112 kb) [file 13041_2018_346_MOESM2_ESM.tif]

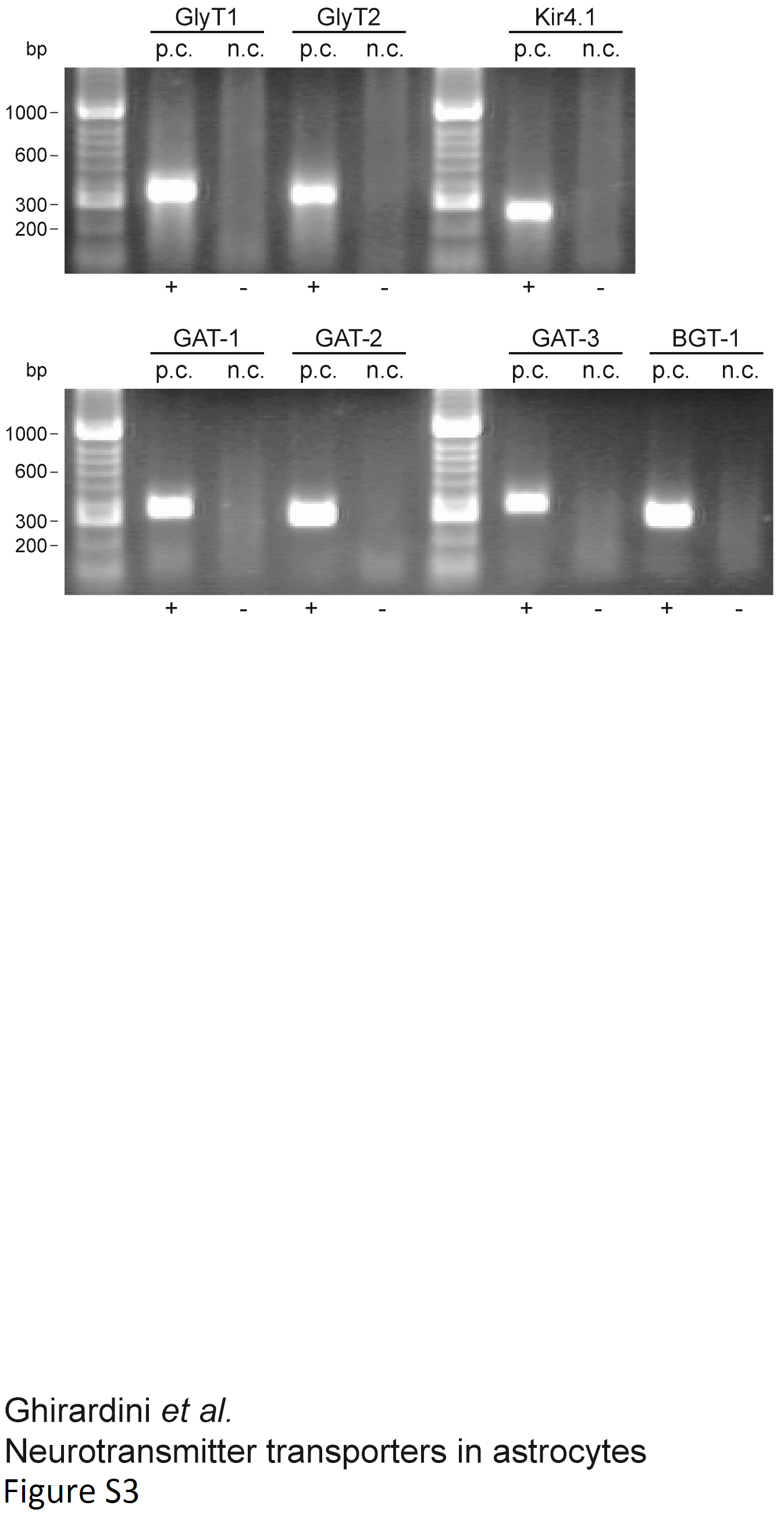

Supplement: Supplementary file 3 — Controls for single-cell RT-PCR. Random RNA prepared from brainstem served as positive control (p.c.). For negative control (n.c.) a patch pipette was dipped into ACSF and was placed closely to the surface of the slice without patching a cell. All targeted mRNAs were detected in p.c., whereas the n.c. was free of signals. (TIFF 1434 kb) [file 13041_2018_346_MOESM3_ESM.tif]
